# Prevalence and Factors Influencing Eye Injuries among Welders in Accra, Ghana

**DOI:** 10.1155/2020/2170247

**Published:** 2020-09-16

**Authors:** Karl Kafui Kwaku Tetteh, Richard Owusu, Wisdom Kudzo Axame

**Affiliations:** ^1^Health Policy Planning and Management Department, School of Public Health, University of Ghana, P.O. Box LG 25, Legon, Greater Accra Region, Ghana; ^2^Epidemiology and Biostatistics Department, School of Public Health, University of Health and Allied Sciences, PMB 31, Ho, Volta Region, Ghana

## Abstract

**Background:**

Eye injuries are one of the most common work-related injuries among certain occupations, including welders. The aim of this study was to determine the prevalence and factors influencing eye injuries among welders in Accra, Ghana.

**Methods:**

In a cross-sectional study, we recruited 382 welders in Accra from two welding sites. Systematic sampling was used to select participants. A pretested semistructured questionnaire was used to collect demographic information, history of eye injuries, ownership, and use of eye protective equipment and workplace characteristics. Bivariate and multivariate logistic regressions at 5% level of significance were used to determine factors influencing eye injuries. Data were entered into Microsoft excel and exported to Stata 16/MP for analysis.

**Results:**

We found 59.7% of welders engaged in electric/arc welding and 40.3% in gas welding. Overall prevalence of eye injuries was 47.9%, higher among electric/arc welders (73.7%) compared to gas welders (9.7%). Factors associated with eye injuries were engaging in gas welding [AOR: 0.08, 95% CI: 0.04–0.16], higher monthly income [AOR = 5.26; 95% CI: 1.72–16.09], nonuse of eye PPE while working [AOR = 1.86; 95% CI: 1.02–3.43], and no training on the use of eye personal protective equipment [AOR = 2.17; 95% CI: 1.07–4.38].

**Conclusion:**

There is high prevalence of welding-related eye injuries among electric welders. Gas welding, high monthly income, nonuse of eye protective equipment, and inadequate training on the use of eye protective equipment were significantly associated with eye injuries. Health policies should be implemented to ensure all welders use eye personal protective equipment.

## 1. Introduction

The eyes are the third most common organ affected by injuries apart from the hands and feet [1]. Eye injuries are common and constitute a major cause of preventable blindness. They are a common cause of visual morbidity occurring at workplaces worldwide [2]. About 2.5 million people succumb to eye injuries annually. Globally, more than 500,000 blinding injuries take place annually [3]. Eye injuries do not occur as random events. Majority of eye injuries have a direct link with occupation and the nature of activity at the time of the injury [1]. Some individuals have high risk of experiencing eye injuries because of their occupations. These individuals include small-scale and large-scale industrial workers. Welding, which involves cutting metal objects, soldering, and in some cases brazing [4], is one of such occupations that pose an exceptional risk to the eye [5]. Though the advent of urbanization and industrialization have brought about automation and mechanization, welding is still an important occupation, particularly in developing countries due to the slow pace of adaption of automated processes in these countries [4]. Industrialization is desirable for all countries. It assures higher standards of living for the citizenry. The welding industry is among the many establishments engaged in the business of producing goods and services. However, welders are susceptible to varied occupational health hazards. Welders form a high risk group for eye injury as a result of exposure to metals and ultraviolet radiations [6]. These exposures are considered to be major risk factors for eye disorders [7].

A study on the evaluation of occupational injuries among welders in Northwest Iran showed that 92% of welders suffered from eye problems [8]. The occupational eye injuries welders experience is because of exposure to ocular hazards. Ocular hazards are elements and circumstances which are a threat to the maintenance and advancement of healthy, comfortable, and wholesome vision [3]. These ocular hazards include welding flash burns, flying metal objects, harmful metal fumes, particulate matter, and thermal burns [2, 9].

Different studies have reported various risk factors associated with eye injuries among welders. A study conducted in Ethiopia stated that poor working conditions, long periods of work, and inadequate safety precautions can lead to increased rates of ocular trauma and disease [10]. A study in India on occupational-related eye injuries reported that workers who were partially trained or have no training when exposed to welding were four times more likely to experience eye injuries compared to a trained worker [5].

The use of appropriate eye protection among welders prevents an estimated 90% of eye injuries [11]. Additionally, the use of appropriate protective eye wear during the welding process has been reported to reduce the harmful effects of infrared, visible, and ultraviolet radiation providing some form of mechanical protection for the eye from weld splatter there by reducing the intensity of visible light during welding [11, 12]. Thus, the benefits of eye devices cannot be overemphasized.

Ghana has a large informal sector which makes up 70% of its workforce [13]. Welding activities generally fall under the informal sector in the country. In Ghana, welders are vulnerable to occupational injuries and accidents because workplace safety is at a very low level due to lack of regulation and inspections, informal management structures, and lack of organizational safety culture [14]. Therefore, injuries associated with this profession could be damaging to the society and economy of the country as these informal sector workers help to mobilize capital and human resources.

Research efforts in relation to occupational health and safety issues in Ghana have been focused on mechanics [15] and woodworkers and the timber industry [16]. There is dearth of research on injuries, particularly eye injuries among welders in the country. Currently, not much is known about eye injuries among welders in Accra. The findings of this study would serve as a basis for advocacy regarding the reduction of eye injuries suffered by welders through welders' education, safety training, and welfare about eye care. This study, therefore, sought to address these identified gaps in research on eye injuries among welders in Accra. Considering the relatively high prevalence of eye injuries reported in some studies, this study is important to identify the prevalence and factors influencing eye injuries among welders in Accra.

## 2. Materials and Methods

### 2.1. Study Design and Setting

The study was a cross-sectional survey which was conducted in March 2019 to determine factors influencing eye injuries among welders in Accra. Our study was conducted at Agbogbloshie and Darkuman Cable and Wireless because they are very busy hubs for informal sector welding activities. These study sites are located in Accra, the capital of Greater Accra Region, Ghana. Agbogbloshie is an informal settlement with considerable overlap between industrial, commercial, and residential zones. It is known as the hotspot of e-waste recycling in the country. Agbogbloshie is separated by Abossey-Okai road and is an extended community as well as one of Ghana's largest urban slums [17]. It serves as home for informal workers and families. Darkuman Cable and Wireless has a mixture of residential buildings, wholesale, and retail shops, formal and informal offices, and workshops, as well as open expanses of informal activities including welding [18].

### 2.2. Sample Size

The required sample size was determined using a formula: *N*=(*Z*^2^pq/*e*^2^) by Cochran [19]. Assumptions were based on reliability coefficient (*z*) of 1.96 at 95% confidence level, margin of error (*e*) of 5%, the proportion (*p*) of 60.2% adopted from a study in Nigeria [9], and *q* = (1 − *p* = 0.398). These figures were substituted into the formula to determine a required sample size for the study. The sample size that was obtained was increased by a nonresponse rate of 5% based on Cochran's formula [19] proportion of population effect. With this, a sample size of 382 was obtained.

### 2.3. Sampling Procedure

Purposive sampling was used to select the two study sites in Accra: Agbogbloshie and Darkuman Cable and Wireless as they are well-known hubs of welding activities in the Accra Metropolitan Area. Proportionate sampling was used to determine the number of welders required at each site to achieve the required sample size. To do this, the total population of welders of the New Korle Lagoon Association of Welders (Agbogbloshie) and National Artisans and Traders Union of Ghana (NATUG), Darkuman Cable and Wireless branch, were used. These totals formed the sampling frame at each study site from which eligible welders were selected. At the study sites, systematic sampling was used to select the welders. To achieve this, a sampling interval *k* was calculated at each site using *N*/*n*, where *N* = population size and *n* = sample size. For Agbogbloshie, *k* = 3, after a random start, every third welder was selected until the calculated sample size was reached. For Darkuman Cable and Wireless, *k* = 5, after a random start, every fifth welder was selected until the calculated sample size was reached. With this, 159 and 223 eligible welders were selected from the New Korle Lagoon Association of Welders (Agbogbloshie) and National Artisans and Traders Union of Ghana (Darkuman Cable and Wireless branch), respectively.

### 2.4. Data Collection

A pretested semistructured questionnaire was used to obtain information on the sociodemographic characteristics, history of eye injury, welding types, and the use of personal protective equipment during welding activities. Data were collected through face-to-face interviews. This process was conducted in either English language or the local language of participants (Twi and Ga). Eligible participants were asked to give informed consent and allowed to participate in the study only after appending their signature or thumb prints to two copies of consent forms. Data were collected during working hours. Interviews were conducted in a safe area at the site away from any distractions.

### 2.5. Quality Control

Before data collection, the questionnaire was pretested outside the study sites, in a similar work environment.

### 2.6. Data Analysis and Management

Questionnaires from the field were coded and checked for consistency before passing on for data entry. Microsoft Excel 2016 was used for data entry. Data was exported to Stata 16/MP for analysis. Our primary analysis for the study was the proportion of welders admitting to have ever suffered any welding-related eye injury. Eye injury was described as any eye trauma, discomfort, or impaired vision as a result of welding activities. Secondary endpoint analysis was focused on age, sex, educational level, number of working years, income, welding type, and the use of eye PPE (independent variables). The use of eye PPE in this study was defined as continuous use of eye PPE such as face shields, impact googles, and general safety glasses while working. A master welder in this study was defined as the owner of a welding shop who supervises other welders in the same establishment and has the most experience on the job.Descriptive statistics was used for continuous and categorical variables.

Univariate and multivariate logistic regression analyses were used with the outcome variable (eye injuries) dichotomized as no = 0 for no eye injury and yes = 1 for eye injuries. The outcome of interest was eye injuries. The strength of associations between independent variables and eye injuries was determined using crude odds ratios (model I). Variables with *p* values of ≤0.2 in model I were considered for a stepwise multivariate logistic regression model (model II). To test for goodness of fit of model II, we used the likelihood ratio test to examine the likelihood of data under the full model as against the likelihood of the data under a model with reduced number of independent variables. We obtained a *p* value for the overall model to be less than 0.05. Thus, we concluded the model was good. Odds ratios and their respective 95% confidence intervals were calculated with *p* values <0.05 considered statistically significant.

## 3. Results and Discussion

### 3.1. Results

#### 3.1.1. Sociodemographic Characteristics of Respondents

Three hundred and eighty-two (382) welders were involved in this study. The mean age of study participants was 32.6 ± 10.96 years (Table 1). Less than half (42.9%) were aged between 20 and 29 years. Majority (97.9%) were males, and 67.8% had primary school education. Few (30.2%) have been in the profession for 1 to 5 years, while 27.2% have been welding for 6 to 10 years. More (45.3%) were masters, and 33.8% were apprentice with 20.9% being workers. Electric/arc welding (59.7%) was the most common welding type. More participants (55.2%) work more than 12 hours daily. Some welders (42.7%) earned between GHS 100 ($18.86) and 500 ($94.34) per month with 18.0% earning more than GHS 1000 per month ($172.46).

#### 3.1.2. Prevalence of Eye Injuries

**Figure 1:**
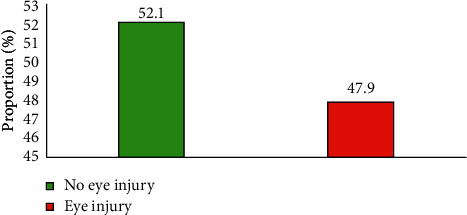
Overall distribution of self-reported eye injuries among welders in the study.

Out of the 382 welders, self-reported eye injuries while welding were 47.9% (95% CI: 42.8%–53.0%), as shown in Figure 1.

#### 3.1.3. Characteristics of Eye Injuries

Among those who experienced eye injuries, a large proportion of the injuries (95.1%) occurred less than a year ago. Most (59.6%) of the injuries sustained occurred in the evening and majority (95.6%) indicated the injuries lasted less than a month. More than half (53.6%) of the injuries were chemical in nature, while only 10.4% were electrical. Most of the welders (83.6%) reported the injuries they sustained affected both eyes (Table 2).

#### 3.1.4. Distribution of Eye Injuries among Welding Types

**Figure 2:**
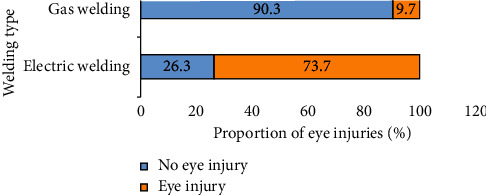
Distribution of self-reported eye injuries among respondents based on welding type.

Eye injuries among electric/arc welders were 73.7%, while only 9.7% of gas welders stated they had ever suffered from eye injuries (Figure 2).

#### 3.1.5. Ownership, Use of Eye PPE, and Workplace Characteristics

Majority (74.1%) of the welders indicated they own an eye PPE. However, usage of eye PPE was 33.2% (Table 3). The most common reason for nonownership of eye PPE was it being not necessary (86.9%). Reasons given for nonuse of eye PPE included the use of eye PPE was not mandatory (58%), eye PPE reduced productivity (22%), and uncomfortable feeling (3.1%). Few (17.5%) of the welders have had training on the use of eye PPE; majority (96.9%) had no workplace eye PPE policy at work and few (3.9%) had safety training at work (Table 3). Almost all (91.1%) stated they were always exposed to nearby welding activities.

#### 3.1.6. Factors Associated with Eye Injuries among Welders

At the bivariate analysis, welding type, monthly income, the use of eye PPE while working, training on use of eye PPE, and exposure to nearby welding activities were associated with risk of eye injuries (Table 4). In the multivariate analysis, gas welders were 92% less likely to have eye injuries compared to their counterparts who engage in electric/arc welding [AOR: 0.08, 95% CI: 0.04–0.16]. Increasing income increased the likelihood of eye injuries. Welders who earn more than GHS 1000 were 5 times more likely to experience eye injuries compared to those who earn less than GHS 100 [AOR: 5.26, 95% CI: 1.72–16.09]. Welders who did not use eye PPE were 86% more likely to experience eye injuries compared to their counterparts who use eye PPE [AOR: 1.86, 95% CI: 1.03–3.33]. Not being trained on how to use eye PPE increased the likelihood of experiencing eye injuries by 2-fold [AOR: 2.17, 95% CI: 1.07–4.38].

## 4. Discussion

This study sought to determine the prevalence and factors influencing eye injuries among welders in Accra. Overall, the study found the prevalence of reported eye injury among welders in Accra to be 47.9%. This is similar to the results obtained in a study conducted in Nigeria by Ihekaire who found the prevalence of eye injury among welders to be 48% [3]. However, this prevalence of is lower compared to 84.5% by Nwala in Nigeria [6], 75% by Ganesh Kumar in India [20], and 61% by Sithole in South Africa [21]. Nwala attributed the high prevalence to increasing industrialization [6], and Sithole attributed their observation to poor knowledge on the adverse effects of welding activities [21]. The prevalence of eye injuries reported in our study could be attributed to the low use of eye PPE. Almost half of welders were found to have suffered from eye injuries. This could have serious socioeconomic consequences for their families, communities, and the country as a whole especially if these injuries are severe and/or permanent, affecting the income generating capability of the welder.

We also found that electric arc welding is the dominant welding type used by welders in Accra (59.7%). A similar pattern was observed in other studies where arc welding was the most common welding type used compared to gas welding [22, 23]. The popularity of arc welding observed in these studies could be attributed to its cost effectiveness, its availability, and ease of use. Among electric welders, 73.7% of them experienced eye injuries, while only 9.7% of gas welders suffered from eye injuries. This agrees with a study by Nartey et al. [23] which found electric welding to be more hazardous. This may be due to the nature of electric welding since it emits a higher ultraviolet light and a brighter flame [24].

The current study found that only 33% of welders used eye PPE while working. This is in agreement with two studies conducted in South Africa [25] and Nigeria [26]. A study reported by Ajayi and Omotoye [27] also reported low usage of eye PPE, as did by Abu et al. [15]. This study reported a higher usage of 33% as compared to that reported by Abu [15] (27.5%). However, this is lower than 47.7% usage of eye PPE which was reported in Nepal [28]. Interestingly enough, 74.1% stated that they own eye PPEs. This is a clear indication that ownership of eye PPE does not necessarily translate into usage. This is also seen in a study by Ajayi which reported that, while 45.9% of welders possessed eye PPE, only 9.6% use eye PPEs all the time [12]. Low use of eye PPE could be attributed to a variety of reasons. Reasons for nonuse identified in this study were reduction in productivity, inconvenience, production of excessive heat, forgetfulness, short duration of task, anticipated low risk of task, and time factor. Some of these reasons are similar to what was reported by Lombardi et al. [29]. These reasons may be categorized broadly as perceptions of hazard and risks, barriers to PPE usage, and enforcement and reinforcement. Another possible factor could be limited knowledge about the importance of PPE. The observed pattern of PPE use among welders in this study suggests an increased risk of eye injuries among welders in Ghana. This is because it has been reported that unsafe occupational acts such as nonuse of PPE are widely associated with injuries [7, 30]. This observation warrants government institutions responsible for Occupational Health and Safety to enforce mandatory use of PPE to ensure welders are more attentive towards personal safety. Without enforcement, eye PPE use is left to the discretion of the employer and employee. Enforcement should be accompanied by positive reinforcement from supervisors and colleagues to ensure the sustainability of eye PPE use. Also, better acceptance of safety procedures will be gained by involving all stakeholders, including workers, by how work tasks should be performed and using the tools necessary to achieve it [29]. The study observed variations in the types of eye PPE used by welders. Most of the eye PPEs were locally manufactured to no set standard. Thus, some of the eye PPEs used were not adequate to help protect against eye injuries. This could have also contributed to the high prevalence of eye injuries.

This study identified a significant association between the nonuse of eye PPE, welding type, monthly income greater than GHS 1000 ($172.46), no training on the use of eye PPE, and eye injuries among welders.

Welders who did not use eye PPE while working were almost twice at risk of eye injuries. This is consistent with similar studies in Nigeria [3] and Nepal [31] where it was reported that nonuse of eye PPE was significantly associated with eye injuries. The primary purpose of any personal protective equipment is to protect the user against health or safety risks at work. In this context, eye PPEs serve as a barrier between the eyes and the external environment. Thus, the use of eye PPE protects the user's eyes from potential chemical, radiological, or mechanical irritants and hazards. Suffice it to say that welders who did not use eye PPE in this study lacked this protection, and this could have contributed to their increased likelihood of experiencing eye injuries.

We also found that gas welders in this study were less likely to experience eye injuries compared to electric/arc welders. An earlier study reported electric/arc welding to be more hazardous than gas welding [32]. Electric welding involves the use of voltage and generates high temperatures which may cause life-threatening injuries. Additionally, continuous exposure to this type of welding can lead to arc eye, a condition in which ultraviolet light inflames the cornea or even burns the retina of the eyes [33]. This could have been the reason why gas welders had reduced odds of welding-related eye injuries.

Our study found that welders with no training on the use of eye PPE were 2 times more likely to experience eye injuries compared to their counterparts with training. Training on the use of eye PPE was provided by the master who has gained experience in the profession over the years to the apprentice and worker welders. These training included the types and correct use of eye PPE while welding. The presence of eye PPE documentations observed in some welding sheds state the continuous use of eye PPE while working also complements master welders in training their subordinates at the workplace. This finding resonates with what was found in India where welders with institutional training on eye PPE were less likely to develop eye injuries [20]. The reason for the reduced odds among welders who have had training on eye PPE could be attributed to increased awareness and knowledge about occupational health and safety [2].

We found that increasing income increased the likelihood of experiencing eye injuries. This finding is similar to a study in Kenya among workers, including welders, which reported that higher income workers take on more jobs and, in their haste to perform these jobs, develop more eye injuries [34]. Also, the higher risk of eye injuries among welders with high monthly income of more than GHS 1000 ($172.46) may be because they undertake more challenging and riskier tasks to earn more money.

## 5. Conclusion

Almost half of the welders have sustained eye injury before. Most of the eye injuries affected both eyes. Majority of welders use electric welding in their activities. Those who use electric welding are more prone to eye injuries compared to gas welders. Usage of eye PPEs is relatively low among welders for a variety of reasons, including the primary reason that usage of eye PPEs is not mandatory. The study found that the welders who do not use eye PPEs have a higher risk of eye injuries. Also, most welders have no form of safety training at work. Increasing income increased the risk of eye injuries.

## 6. Recommendations

Considering the importance of eye PPEs in the prevention of eye injuries, the use of eye PPEs by welders should be increased through education and training on the use of eye PPE, making the use of eye PPE mandatory. These should be implemented through the relevant welder associations. Welding associations should prioritize interventions to reduce eye injuries particularly among electric welders since they form the majority of welders who suffer more eye injuries. There is also a need to conduct further studies using both quantitative and qualitative methods to include all kinds of welders to obtain results generalizable to the welding community.

## 7. Limitation

Despite the important findings made in our study, it is worth noting the possible limitations inherent in its conduct. Our results were premised on responses provided by participants based on events which occurred in the past (though not too distant). There is therefore the possibility of recall bias. Our study did not include other types of welders such as industrial welders in order to generalize findings to the welding community. It is however important to note that these potential limitations do not affect the validity and reliability of our findings in any way.

## Figures and Tables

**Table 1:** Background characteristics of welders (*N* *=* *382*).

Characteristics	Frequency	Percentage (%)
Age (years), mean (±SD)	32.6 (±10.96)	
<20	23	6.0
20–29	164	42.9
30–39	103	27.0
49–49	54	14.1
50–59	27	7.1
≥60	11	2.9

Sex		
Male	374	97.9
Female	8	2.1

Education		
No formal education	31	8.1
Primary	259	67.8
Secondary	83	21.7
Tertiary	9	2.4

Working experience (years), mean (±SD)	10.1 (±8.65)	
<1	20	5.2
1–5	115	30.2
6–10	104	27.2
>10	143	37.4

Type of welder		
Apprentice	129	33.8
Worker	80	20.9
Master	173	45.3

Welding type		
Electric/arc welding	228	59.7
Gas welding	154	40.3

Daily working hours		
<12 hours	171	44.8
≥12 hours	211	55.2

Monthly income (GHS)		
<100	66	17.3
100–500	163	42.7
600–1000	84	22.0
>1000	69	18.0

**Table 2:** Characteristics of eye injuries among welders (*N* = 183).

Variable	Frequency	Percentage (%)
Period of last eye injury		
Less than a year ago	174	95.1
More than a year ago	9	4.9

Time of day of last injury		
Morning	35	19.1
Afternoon	39	21.3
Evening	109	59.6

Duration of eye injury		
Less than a month	175	95.6
More than a month	8	4.4

Nature of eye injury		
Mechanical	39	21.3
Chemical	98	53.6
Thermal	27	14.7
Electrical	19	10.4

Number of eyes affected		
One eye	30	16.4
Both eyes	153	83.6

**Table 3:** Ownership, use of eye PPE, and workplace characteristics.

Variable	Frequency	Percentage (%)
Own eye PPE		
No	99	25.9
Yes	283	74.1

Reasons for nonownership of eye PPE		
Not necessary	86	86.9
Too expensive	8	8.1
Do not know where to get one	5	5.0

Usage of eye PPE while working		
Nonuse	255	66.8
Use	127	33.2

Commonly used eye PPE		
Glasses	57	44.9
Goggles	51	40.2
Eye shields	14	11.0
Others	5	3.9

Reasons for nonuse of eye PPE		
Use of eye PPE not mandatory	148	58.0
Reduces productivity	56	22.0
Low risk of task	22	8.6
Feels uncomfortable	8	3.1
In a hurry	8	3.1
Produces heat	5	2.0
Forgetfulness	5	2.0
Short duration of task	3	1.2

Training on use of eye PPE		
No	315	82.5
Yes	67	17.5

Workplace eye PPE policy		
Present	13	3.4
Absent	369	96.6

Safety training at work		
Never	301	78.8
Sometimes	66	17.3
Often/always	15	3.9

Exposure to nearby welding activities		
Never	12	3.1
Sometimes	22	5.8
Often/always	348	91.1

**Table 4:** Factors associated with eye injuries among welders.

Variable	Eye injury *n* (%)	Model I COR		Model II AOR	
		OR (95% CI)	*p* value	OR (95% CI)	*p* value
Age (years)			0.3813		
<20	9 (4.9)	1			
20–29	81 (44.3)	0.66 (0.27–1.60)			
30–39	42 (22.9)	0.93 (0.37–2.35)			
49–49	31 (16.9)	0.48 (0.18–1.29)			
50–59	14 (7.6)	0.60 (0.19–1.84)			
≥60	6 (3.3)	0.54 (0.12–2.29)			

Educational level			0.6860		
No formal education	13 (7.1)	1			
Primary	125 (68.3)	1.29 (0.61–2.74)			
Secondary	42 (22.9)	1.42 (0.62–3.26)			
Tertiary	3 (1.6)	0.69 (0.15–3.29)			

Working experience (years)			0.6450		
<1	8 (4.4)	1			
1–5	56 (30.6)	0.70 (0.27–1.85)			
6–10	46 (25.1)	0.84 (0.32–2.23)			
>10	73 (39.9)	0.64 (0.25–1.66)			

Welding type			<0.001		
Electric/arc welding	168 (91.8)	1		1	
Gas welding	15 (8.2)	0.04 (0.02–0.07)		0.08 (0.04–0.16)	<0.001^*∗*^

Monthly income (GHS)			0.2064		
<100	32 (17.5)	1.		1	
100–500	82 (44.8)	1.07 (0.61–1.90)		1.27 (0.60–2.70)	0.529
600–1000	32 (17.5)	0.65 (0.34–1.26)		1.49 (0.58–3.79)	0.407
>1000	37 (20.2)	1.23 (0.62–2.41)		5.26 (1.72–16.09)	0.004^*∗*^

Usage of eye PPE while working			0.0877		
Use	53 (29.0)	1			
Nonuse	130 (71.0)	1.45 (0.944–2.23)		1.86 (1.03–3.33)	0.040^*∗*^

Training on use of eye PPE			0.1867		
Yes	37 (20.2)	1		1	
No	146 (79.8)	1.43 (0.84–2.42)		2.17 (1.07–4.38)	0.030^*∗*^

Eye PPE policy at work			0.009		
Present	12 (6.6)	1		1	
Absent	171 (93.4)	9.64 (1.75–53.10)		0.05 (0.01–1.39)	0.077

Safety training at work			0.811		
Never	142 (77.6)	1			
Sometimes	34 (18.6)	1.19 (0.70–2.03)			
Often/always	7 (3.8)	0.98 (0.35–2.77)			

Exposure to nearby welding activities			0.0001		
Never	3 (1.6)	1		1	
Sometimes	2 (1.1)	0.3 (0.04–2.11)		0.08 (0.01–1.08)	0.057
Often/always	178 (97.3)	3.144 (1.84–11.80)		1.22 (0.23–6.58)	0.813

^*∗*^Statistically significant at *p* value <0.05.

## Data Availability

All data and materials are available upon reasonable request from the corresponding author.

## Abbreviations

PPE: Personal protective equipment
NATUG: National Artisans and Traders Union of Ghana
AOR: Adjusted odds ratio
GHS/ERC: Ghana Health Service Ethics Review Committee.
